# Creative brains: designing in the real world^†^

**DOI:** 10.3389/fnhum.2014.00241

**Published:** 2014-04-30

**Authors:** Vinod Goel

**Affiliations:** ^1^Department of Psychology, York UniversityToronto, ON, Canada; ^2^Department of Psychology, University of HullHull, UK

**Keywords:** architecture, creativity, drawing, representations, cognition, neuropsychology, lesion studies, hemispheric asymmetry

## Abstract

The process of designing artifacts is a creative activity. It is proposed that, at the cognitive level, one key to understanding design creativity is to understand the array of symbol systems designers utilize. These symbol systems range from being vague, imprecise, abstract, ambiguous, and indeterminate (like conceptual sketches), to being very precise, concrete, unambiguous, and determinate (like contract documents). The former types of symbol systems support associative processes that facilitate lateral (or divergent) transformations that broaden the problem space, while the latter types of symbol systems support inference processes facilitating vertical (or convergent) transformations that deepen of the problem space. The process of artifact design requires the judicious application of both lateral and vertical transformations. This leads to a dual mechanism model of design problem-solving comprising of an associative engine and an inference engine. It is further claimed that this dual mechanism model is supported by an interesting hemispheric dissociation in human prefrontal cortex. The associative engine and neural structures that support imprecise, ambiguous, abstract, indeterminate representations are lateralized in the right prefrontal cortex, while the inference engine and neural structures that support precise, unambiguous, determinant representations are lateralized in the left prefrontal cortex. At the brain level, successful design of artifacts requires a delicate balance between the two hemispheres of prefrontal cortex.

## INTRODUCTION

**FIGURE 1:**
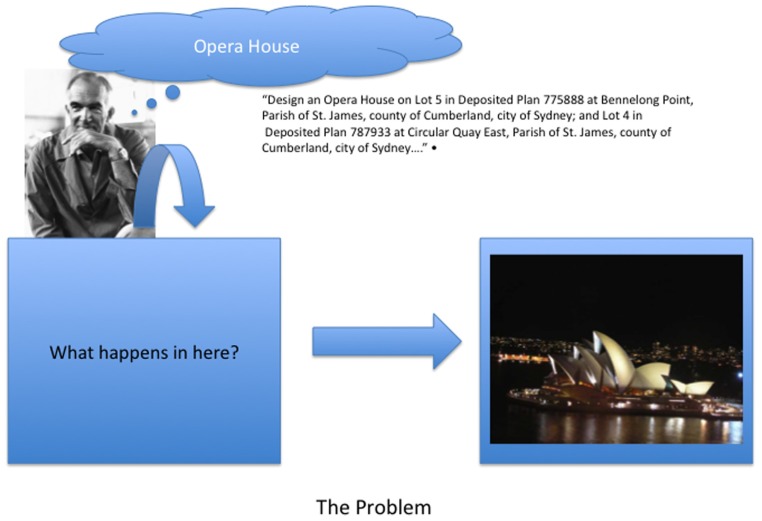
**The input to the designer is a design brief, the output are the contract documents specifying the artifact.** The problem of interest is what cognitive and neurological structures and processes support the transformation of the input to the output.

The central problem of designing and creating artifacts can perhaps be captured in the example in **Figure 1**. A young architect, Jorn Utzon, is confronted with the problem of designing an opera house for a site in Sydney, Australia and generates the drawings for specifying the artifact depicted in **Figure 1**. No other animal is capable of doing this. The question of interest is what cognitive and neurological processes make it possible?

There are at least two popular accounts of how this act of creation comes about. The first is the *ex nihilo* account from Genesis. “And God said let there be light...” (or an opera house) and it was thus. It is a simple, straightforward account with broad appeal. Throughout literature poets and artists have been invoking Gods to inspire their works, as does Homer in the first few lines of the Iliad (“Mênin aeide, thea, Pêlêiadeô Achillêos....”). The only problem is that one needs to be a God (or at least inspired by God) to create *ex nihilo*. Presumably, this route was not available to Utzon.

The second account is more earthly. It is a process of combining existing ideas and concepts in a manner useful for an intended purpose (Koestler, 1975). On this “select and combine” account, Utzon does not begin with a blank slate. He has considerable knowledge about the world, including opera houses. All he has to do is to select and modify existing structures to suit his specific conditions. Indeed, in his retrospective account of the process he notes being inspired by memories of the Castle of Kornborg, the Yucatán Peninsula in Mexico, and seeing the naval charts over Sydney^1^.

This latter view of creation is endorsed by many. For example, Wittgenstein (1993, p. 228) noted that “problems are solved, not by giving new information, but by arranging what we have known since long.” More recently, Steve Jobs stated “creativity is just connecting things. When we ask creative people how they did something, they feel a little guilty because they did not really do it, they just saw something. It seemed obvious to them after a while. That’s because they were able to connect experiences they’ve had and synthesize new things. And the reason they were able to do that was that they’ve had more experiences than other people^2^.” I think this viewpoint, at best, begs several critical questions. If nothing else, it demonstrates that being creative does not necessarily give one insight into the process of creativity.

**FIGURE 2:**
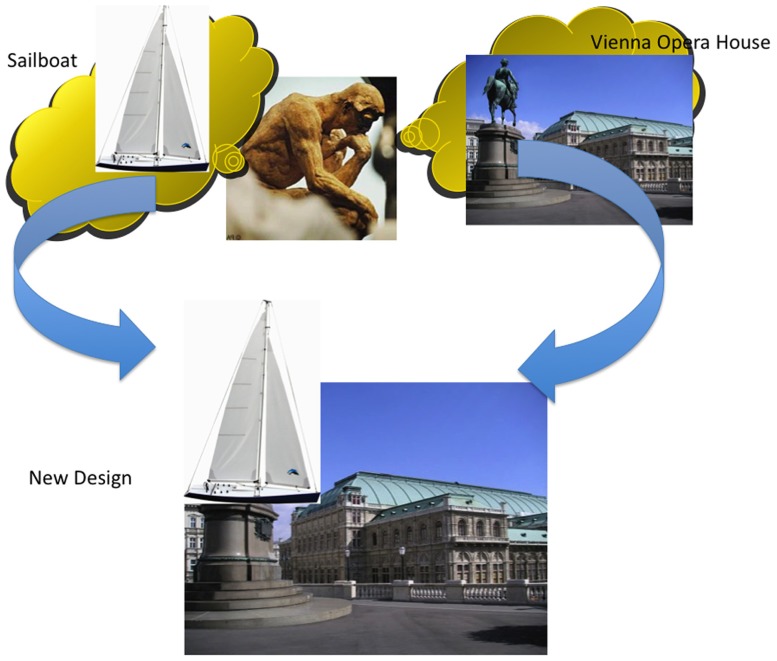
**The select-and-combine model of creativity.** The above selection and combination are consistent with the select-and-combine model, and serve to highlight its inadequacy.

For example, confronted with the same task as Utzon, I might, following the select-and-combine model proceed as follows: I have some knowledge of the world and existing opera houses. I also know that the site of the proposed opera house is on the harbor. So perhaps I can select elements from existing opera houses and combine them with elements from nautical themes. (There is certainly a deeply entrenched story within the architecture community about how Utzon came up with the basic idea by watching and drawing sailboats in the harbor.) I could start with the famous opera house in Vienna and a sailboat, and combine them as in **Figure 2**. In doing so, I’m proceeding as prescribed by the select-and-combine model of creativity.

However, the ludicrous result, depicted in **Figure 2**, highlights two critical shortcomings of the select-and-combine model. The first issue is one of determining relevant or salient features and elements from irrelevant or non-salient features and elements. The second issue is that the selection and combination of features and elements requires abstraction and modification. They cannot simply be cut and pasted from one context to another. The first problem is an instance of the ubiquitous problem of induction, often known as the “Frame Problem” in the cognitive science literature. It is pervasive throughout cognitive psychology and is present in the framework developed here. The main focus of this article is to present some ideas for dealing with the second issue in the context of architectural design.

There is a literature in cognitive psychology that focuses on creativity. I will begin by suggesting that this literature, while relevant, misses the mark, or is at least incomplete, in terms of understanding creativity in the design process. An underlying theme of the article is that creativity is probably not a unitary concept to be identified and exposed. It is more likely a byproduct of real-world problem-solving, as suggested by the select-and-combine model of creativity (see also Weisberg, 1993). I will suggest that one key to understanding design creativity is to appreciate the array of symbol systems designers utilize. These symbol systems range from being vague, imprecise, abstract, ambiguous, and indeterminate (like conceptual sketches) to being very precise, concrete, unambiguous, and determinate (like contract documents^3^). These symbol systems are essential in allowing for the abstractions and modifications required by the select-and-combine model of creativity. The former types of symbol systems support associative mechanisms facilitating lateral (or divergent) transformations that broaden the problem space, while the latter types of symbol systems support inference mechanisms that facilitate vertical (or convergent) transformations that deepen the problem space (Goel, 1995). This results in a dual or multiple mechanism model of problem solving, not unlike that proposed by Sloman (1996) for logical reasoning. These two systems work together in successful design problem solving. Furthermore, there is evidence of hemispheric dissociations in prefrontal cortex (PFC) corresponding to the different cognitive representations and mechanisms. The last section will present evidence that the right PFC tolerates imprecise, ambiguous, abstract, indeterminate representations, and is more suited for lateral transformations while the left PFC supports precise, unambiguous, determinant representations, and facilitates vertical transformations.

## RELATING THE COGNITIVE CREATIVITY LITERATURE TO THE DESIGN PROCESS

There is a large cognitive psychology literature on creativity (Barron and Harrington, 1981; Hocevar, 1981; Sternberg, 1999; Jung-Beeman, 2005; Vartanian, 2009; Kaufman and Sternberg, 2010). But, the constraints of experimental design largely preclude studies embedded in real world contexts, such as design problem-solving (though see Mansfield and Busse, 1981; Gardner, 1982; Weisberg, 2011). [There is a literature on design creativity within the design and engineering disciplines (Gero and Maher, 1993; Dorst and Cross, 2001; Goldschmidt and Tatsa, 2005), but it lies beyond the scope of this review]. Creativity-related research in cognitive psychology seems to have two primary foci. One focus is the literature on insight/“aha” problems; the second is the literature on divergent thinking tasks.

### INSIGHT PROBLEMS

Examples of insight problems include the candlestick problem, triangle problem, and the radiation problem, among others (Katona, 1940; Duncker, 1945). These types of problems are widely characterized by an impasse (no obvious solution), fixation (repetition of the same types of unsuccessful steps), incubation (disengagement of the problem), and a sudden solution (“aha” experience; Duncker, 1945).

**FIGURE 3:**
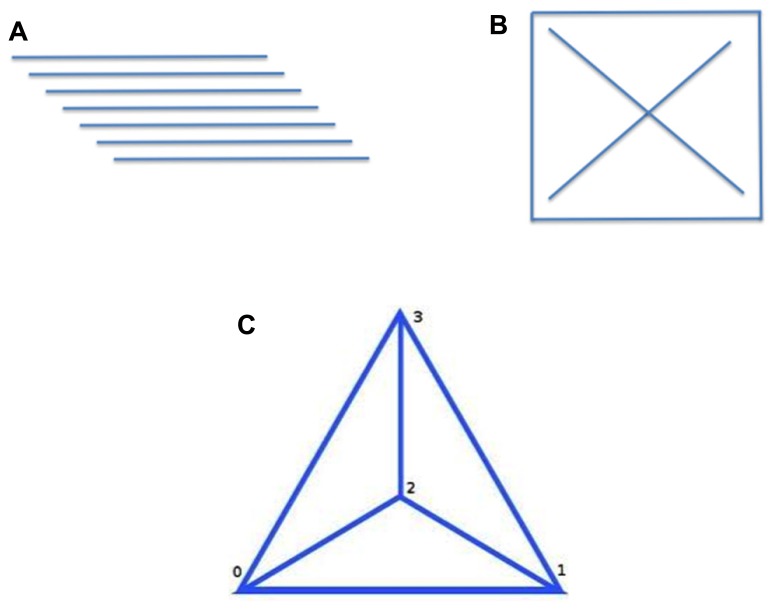
**The triangle problem** (Katona, 1940). The goal of the task is to arrange six sticks of equal length **(A)** into four equilateral triangles. **(B)** While one can form four triangles in two-dimensional space, they will not be equilateral. **(C)** Once the third dimension is considered, the solution presents itself.

Consider the triangle problem task in **Figure 3**. The task is to arrange six sticks of equal length (**Figure 3A**) to form four equilateral triangles. Most participants look for a solution within a two-dimensional problem space (**Figure 3B**). Four triangles can indeed be formed in this manner, but they will not be equilateral triangles. The problem has no two-dimensional solution. There is nothing about the problem constraints that specifies a two dimensional solution. It seems to be an implicit, self imposed constraint. However, once participants suppress or relax this constraint and explore three-dimensional representations of the problem, the solution presents itself (**Figure 3C**). Interestingly, the 3D space provides exponentially more possibilities for arranging the six sticks, but nonetheless, the solution seems fairly obviously, perhaps restricted by the affordances of the problem.

Most insight problems are quite removed from design problems in that they are a special subset of well-structured problems (Reitman, 1964; Goel, 1995). While there is much to be said about the relationship between well-structured and ill-structured problems (Goel, 1995), here I will focus on two points: (1) well-structured problems have completely specified start states, goal states, and transformation functions that map the former onto the latter whilst these critical components in ill-structured problems are incompletely specified (Reitman, 1964); and (2) the constraints of well-structured problems are constitutive of the task while the constraints on ill-structured problems are flexible and negotiable (Goel, 1995). Real-world problems (like design problems) have both, ill-structured and well-structured components.

As the triangle problem is a well-structured problem, it is relatively easy to specify a problem space. The start state in the triangle problem is completely and unambiguously characterized by the given six sticks of equal length (**Figure 3A**). The goal state is to arrange them in four equilateral triangles. The transformation function is also specified and on the surface seems trivial – rearrange the sticks. The task is to apply this transformation function in a generate-and-evaluate cycle to come up with a pattern that satisfies the goal state. The task, despite being well-structured, may, of course, be non-trivial.

Insight problems differ from the broader class of well-structured problems in that the goal state lies in a part of the problem space that is unconnected (or remotely connected) to, or not “visible” from the current state of the problem solver. The phenomenological experience of the problem solver is one of being suddenly transferred from the current node in the problem space to a node that is connected to or near the goal state. Once this mental set shift or reconceptualization occurs, the problem solver can access the goal state using standard “convergent thinking” processes.

### DIVERGENT THINKING OR SEMANTIC SPREAD TASKS

Divergent thinking or semantic spread tasks are the second widely used class of creativity tasks in the cognitive psychology literature. The Alternative Uses Task (Guilford, 1967) provides a good example of such problems. The goal in the Alternative Uses Task is to generate as many uses as possible of familiar objects (e.g., hammer, stapler, rock, etc.). It is argued that success in this task is a function of “defocused” attention (Vartanian, 2009). The more defocused a subject’s attention, the more widely dispersed connections they will make.

This category of tasks differ from insight problems in at least three important ways: (i) they are a subset of ill-structured problems; (ii) they involve continuous divergent transformations (as opposed to a discreet mental set shift, followed by convergent transformations); (iii) there is no sudden “aha” experience associated with discovery of the goal state. Solutions are gradually developed^4^.

### COGNITIVE THEORIES OF CREATIVITY

In terms of cognitive theories of creativity, there are two, slightly different accounts. On one account, represented by the works of Kaplan and Simon (1990), Ormerod et al. (2002), and Ward et al. (1998), there is nothing special about creativity. Creativity tasks simply place differential demands on memory and attentional systems.

The second account, directed at insight problems, as presented by Ohlsson (1992) and Öllinger and Knoblich (2009) focuses on representational change through chunk decomposition and/or constraint relaxation. They hold that people make implicit assumptions based upon properties of the representation of the problem, that in turn hamper solutions. An example is provided by the triangle problem in **Figure 3**. Participants assume a two-dimensional solution space, even though nothing in the problem statement requires this. As already noted, the problem has no two-dimensional solution. However, once participants form a three-dimensional representation of the problem space, the solution “presents itself.” On both accounts creativity tasks are accommodated within the standard cognitive information processing theory framework (Newell and Simon, 1972).

My concern is with accounting for creativity within the architectural design process. I will also argue for the critical importance of representational change. However, the account I will advocate differs from both of these accounts in that the type of representational change that I believe is required for real-world problem solving necessitates the use of a wide range of qualitatively different symbol systems, whereas, the examples in this literature always involve a transformation within the same symbol system (Goel, 1995). As many symbol systems result in vague, ambiguous, indeterminate representations, they lie beyond standard information processing theory models of cognition (Goel, 1995).

### LIMITATIONS OF THE CREATIVITY LITERATURE IN UNDERSTANDING DESIGN ACTIVITY

I want to suggest that a focus on insight problems and divergent thinking problems, while capturing genuine issues in terms of creativity, may not be particularly germane or central to our problem of understanding creativity in the real world context of designing artifacts.

There are two important sources of discontinuity between classic insight problems and real world design problems. First, the design of artifacts has both ill-structured and well-structured components, while insight problems are usually well-structured. This has enormous consequences for the structure of the problem space (Goel, 1995). Second, while there may have been a *eureka* moment on Utzon’s route to the design of the opera house, the design certainly did not emerge, fully formed. The basic idea would have evolved over a period of days to months.

There may be a more natural connection between divergent thinking tasks and the design of artifacts, but even here the connection is incomplete. A defocused mind, on its own, is not a productive mind. While divergent thinking (or making connections between widely dispersed concepts) is critical for design, it is not sufficient. Design also requires convergent thinking. For example, while “hammering a nail” may be one possible use of a rock, in actuality it may not be practical/suitable unless it is shaped in a certain way or fastened to a handle, etc.

The value of the cognitive creativity literature is to highlight the importance of divergent/lateral transformation (defocused attention) mechanisms and representational change as necessary components of creativity. It’s shortcomings are (1) ignoring the equally important role of convergent/vertical transformations in real-world creativity tasks; (2) ignoring the distinction between well-structured and ill-structured problems, and (3) not appreciating the critical role of the structure of symbol systems in facilitating divergent and convergent transformations. The next section expands on these three themes and endorses a dual mechanism model of design problem- solving.

## COGNITIVE CHARACTERIZATION OF THE DESIGN PROCESS

This analysis begins by making two common assumptions (Chomsky, 1975; Fodor, 1975; Newell, 1980): (i) that all thought processes are goal directed transformations of mental representations; (ii) an analysis of the structure of external representations used by problem solvers gives us an insight into the structure of their internal mental representations and transformations. These assumptions commit us to a central role for representations and restrict the space of explanations. Within this framework we can appeal to representational structures and the (computational) procedures that transform the representations^5^. The two are, of course, not unrelated. Every representational structure will facilitate certain types of transformations/computations and hinder others. For example, list-type data structures facilitate the computation of certain functions (e.g., sorting a list of items), while matrices-type data structures facilitate the computation of other functions (e.g., image processing).

**FIGURE 4:**
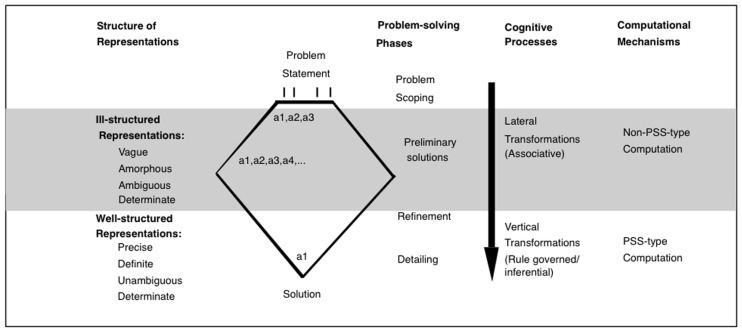
**Aspects of design problem solving.** Unlike the state space for well-structured problems, the state space for ill-structured, real-world problems must support different problem-solving phases, which need to be supported by different representational systems, cognitive processes, and computational mechanisms. PSS-type and non-PSS-type indicate physical symbol system type mechanisms (Newell, 1980) and non-physical symbol system type mechanisms, respectively. A strict dichotomy of representational systems and processes is not intended. The intention is to convey a spectrum of representational systems (and processes) along the dimension of indeterminate and determinate.

Design problem-solving is typically characterized as a multistep process involving problem scoping/framing, generation of preliminary ideas, refinement, and detailing. Each phase differs with respect to the type of information dealt with, the degree of commitment to generated ideas, the level of detail attended to, the number and types of transformations engaged in, the mental representations needed to support the different types of information and transformations, and the corresponding computational mechanism (Goel, 1995). As one progresses from the preliminary phases to the detailing phases, the problem becomes more structured. This is depicted in **Figure 4**.

Problem scoping/framing is the process of interpretation of an open-ended problem statement in the context of the designer’s world knowledge, goals, aspirations, abilities, resources, etc. The initial framing of the problem is a function of this poorly understood interpretation process rather than the original problem statement.

Preliminary solution generation is a classic case of creative problem solving. It is a phase of “cognitive way-finding”, a phase of concept construction, where a few kernel ideas are generated and explored through lateral transformations. A lateral transformation is one where movement is from one idea to a slightly different idea rather than a more detailed version of the same idea. Lateral transformations are necessary for the widening of the problem space and the exploration and development of kernel ideas. This generation and exploration of ideas/concepts is facilitated by the abstract nature of information being considered, a low degree of commitment to generated ideas, the coarseness of detail, and the number of lateral transformations.

The refinement and detailing phases are more constrained and structured. They are phases where preconstructed concepts are manipulated. Commitments are made to a particular solution and propagated through the problem space. They are characterized by the concrete nature of information being considered, a high degree of commitment to generated ideas, attention to detail, and a large number of vertical transformations. A vertical transformation is one where movement is from one idea to a more detailed version of the same idea. It results in a deepening of the problem space. The rules underlying vertical transformations can often be articulated (Goel, 1995).

The ability to engage in lateral transformations is underwritten by a mechanism that supports ill-structured mental representations. Ill-structured representations are imprecise, ambiguous, fluid, indeterminate, vague, etc. The ability to engage in vertical transformations is underwritten by a mechanism that supports well-structured mental representations and computation. Well-structured representations are precise, distinct, determinate, and unambiguous. It has been further argued that there is a computational dissociation between these two mechanisms (Goel, 1995; Giunti, 1997). Ill-structured and well-structured representations differ with respect to formal properties. This in turn affects the modes of inference they can participate in and the computational mechanisms required to support them.

**FIGURE 5:**
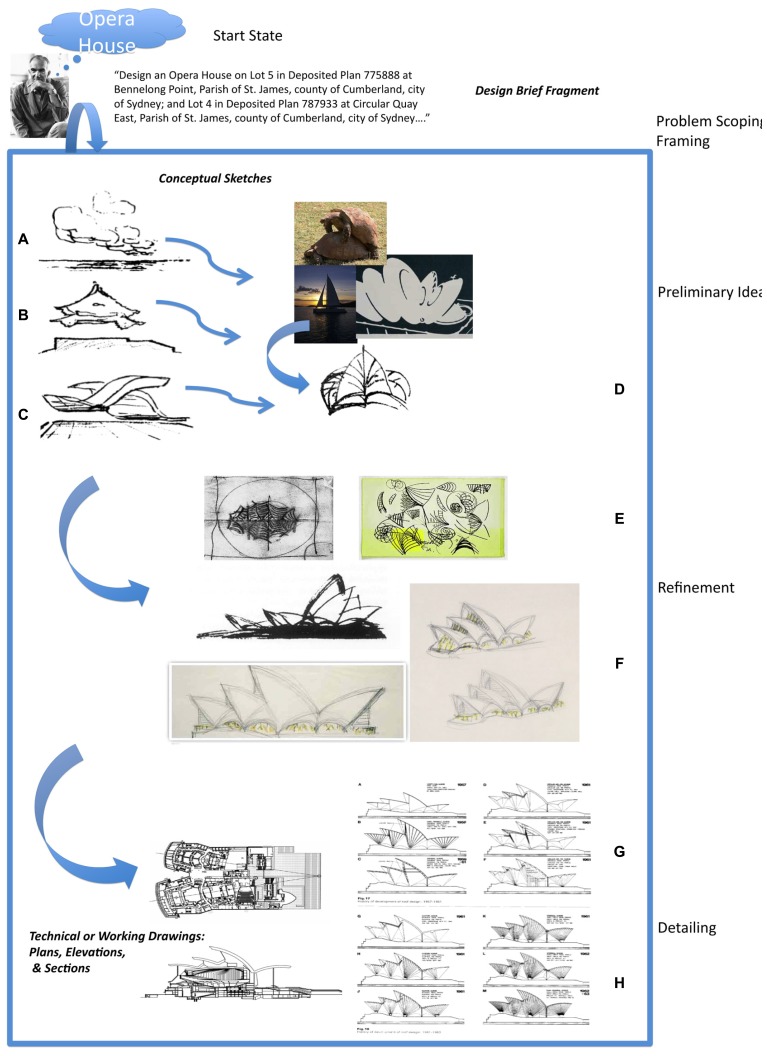
**An incomplete and highly speculative reconstruction of Utzon’s problem space.** One crucial key to understanding the design problem space is to look at the nature of the several different types of representations utilized by designers. They can be broadly divided up into natural language, conceptual sketches, and contract documents. Design briefs consist largely of natural language sentences. Their level of precision and ambiguity varies. Drawings **A**–**D** are examples of early conceptual sketches. There is often no fact of the matter as to what they represent. The “what is that?” is often discovered and emerges after the drawing. Drawings **E** and **F** show the development of one of the ideas introduced in the conceptual sketches. The artifact is beginning to take a specific form and starting to be fleshed out. Drawings **G** and **H** are examples of technical drawings or blueprints that will form part of the contract documents. They specify the artifact in a very precise, complete, unambiguous, and determinate manner. The differences between the conceptual sketches and the working drawings (ostensibly both “pictorial”) are at least as great as the differences between the design brief and the conceptual sketches. See text.

**Figure 5** depicts a highly incomplete and speculative reconstruction of Utzon’s problem space. The start state is the design brief. The goal state is something as vague as “design an Opera House for this site on the Sydney Harbor….” The intermittent states (incomplete) are the depicted drawings, the outcomes of transformations from the design brief to the preliminary sketches, through refinement, to the detailed drawings. Unlike the case of the triangle problem task (**Figure 3**), the task here is not one of discovering a path from a start state to a goal state in a problem space specified by given constraints. The problem space does not come prespecified. It must be constructed on route. In fact, it can be argued that the specification of the problem space is the problem (Rittel and Webber, 1974). While I will continue to use the term “problem space” in the context of design (for the lack of a better term), it should be clear that I believe that there is a discontinuity in the meaning of this term in the context of the triangle problem task (well-structured problem) and a design task (real-world problem). The reader is referred to Simon (1973) for a contrary view.

Our present focus is on the nature of the representations and transformations that we find in the designer’s problem space, and how they evolve over time.

The design brief is a natural language document. It has a reasonably “precise” meaning but clearly allows for enormous latitude, multiple interpretations, and negotiation (i.e., the constraints it provides are not constitutive of the task). This can be seen in the fact that any three different architects would interpret the design brief in three different ways and generate three different start states (to say nothing of the actual design/artifact).

The initial drawings made by designers are much less precise and much more abstract than the actual design brief. They are indeterminate, vague, and ambiguous. In fact, there need not be any fact of the matter as to what they represent. Does **Figure 5D** depict a sailboat, copulating turtles, stacked plates? All of the above? None of the above? The situation that designers find themselves in here is not unlike Wittgenstein’s (1993, p.193) philosophers who like little children “first scribble random lines on a piece of paper with their pencils, and now ask an adult ‘What is that?’.” The “what is that” is often discovered and emerges from the conceptual drawings after the fact.

As the designer moves from these preliminary/conceptual drawings to more detailed drawings (**Figures 5E,F**), the indeterminacy and ambiguity begin to dissipate. (This will rarely be a linear process. The designer will reiterate between indeterminate and determinate representations several times.) By the time the final drawings are generated (**Figures 5G,H**), the representation of the artifact is determinate. There is now a one-to-one mapping between the representations (drawings and specifications) and the to-be-constructed artifact.

This process of moving from an ill-defined scenario to more abstract, indeterminate representations and then back to more determinate representations is depicted in **Figure 5**. We might summarize the relationship between the design brief, preliminary sketches, and contract documents as follows: contract drawings are determinate and allow for the representation of articulate, precise, concrete, and unambiguous informational states. Conceptual sketches are indeterminate and allow for the representation of vague, inarticulate, imprecise, abstract, and ambiguous informational states. Design briefs fall somewhere between these two extremes.

Not only do designers use several different types of symbol systems, we find that each system is used at a particular time, and for a particular purpose. I don’t believe this is an accident. It is an outcome of the fact that *the structure of symbol systems constrains the message/information that it can encode* (Goodman and Elgin, 1988). This tight coupling between the structure of symbol systems and informational content also implies that internal and external symbol systems must share similar properties to encode similar information.

In addition to the nature of the representations, there are the cognitive transformations to consider. The initial transformations, particularly from the design brief to the preliminary sketches, and among the preliminary sketches, correspond to the above-mentioned defocused attention or divergent/lateral transformation processes. Notice the relationship between the design brief and the initial drawings. There is a very explicit process of abstraction that occurs here. The drawings are somehow connected to, but not entailed by the design brief. For example, what in the design brief entails the connection to the Yucatán Peninsula in Mexico that Utzon reports making (Footnote 1)? The connections here are some form of whimsical, serendipitous associations (conceptual, semantic, economic, esthetic, resemblance, etc.). Many different instantiations would be consistent with the design brief, and several different ones may be considered. Similarly, the relationships between the early conceptual sketches are also associative, as opposed to inferential. (See Fodor and Pylyshyn (1988) for a discussion of the distinction between inference and association]. As one moves through the problem space and the drawings become more determinate, the transformations begin to correspond to convergent/vertical processes, which are amenable to inference relationships. (e.g., if the building code requires three fire doors for the design you have developed, then you must provide three fire doors… You may of course try to negotiate this or reconceptualize the design, but that just pops you up to an earlier phase in the process).

The whole process is, of course, carried out in the context of expert knowledge of architectural design. The use of indeterminate representations and vague associations in the early explorative phases of the design process are explicit tools used to move beyond the borders of expert preconceptions. Technical knowledge and skill is more prevalent in the more constrained, latter half of the process.

A formal and more complete discussion of the structure of the symbol systems used by designers and how they facilitate design problem-solving is offered elsewhere (Goel, 1995). For present purposes the main point is that real-world design problem-solving requires at least two different types of transformation processes, variously referred to as divergent/lateral and convergent/vertical transformations. The former broaden the problem space while the latter deepen it. Both are necessary. These two different types of transformations are facilitated by different types of representations. While there are several differences in the various representations used by designers (see Goel, 1995), in the above discussion I have focused on one such difference: the variability along the dimension of determinacy. Divergent thinking processes are defined over largely indeterminate representations while convergent thinking processes benefit from determinate representations.

An important outcome of the more complete, formal analysis is that indeterminate representations, while necessary for real-world problem solving, are inconsistent with standard information processing theory models of problem-solving (Goel, 1995). A dual mechanism model of real-world problem-solving is required. One component of this model can be captured by standard information processing theory accounts (Newell and Simon, 1972; Newell, 1990). The other component requires a different type of mechanism, perhaps something akin to the “subsymbolic”/associative models (Smolensky, 1988). Both mechanisms are required to account for real-world problem solving, such as design. In the next section I review neuropsychological evidence supporting this position.

## BRAIN SYSTEMS INVOLVED IN DESIGNING ARTIFACTS

### FRONTAL LOBE LATERALIZATION HYPOTHESIS

I have argued for a dual mechanism cognitive account of real-world problem-solving. The question now is whether there is any support for such a model at the neuropsychological level. I will suggest that there is evidence for anatomical dissociations corresponding to the cognitive dissociations identified in the previous section. To this end I propose the Frontal Lobe Lateralization Hypothesis (FLLH).

FLLH: Left and right prefrontal cortex make differential contributions (in terms of the structure of representations and types of transformations) to real-world problem solving. The right PFC supports abstract, vague, ambiguous, indeterminate representations of the world, while the left PFC abhors uncertainty and tries to automatically fill in the gaps with concrete, determinate, unambiguous, specific information. The system is set up in such a way that each hemisphere also tries to inhibit the other, though usually the left dominates (see below). Successful functioning in the real world is a judicious balancing act between these two systems. Damage to either system will result in impaired real-world performance, but with different cognitive signatures. Damage to right PFC system will allow the left PFC free reign to prematurely lock on to patterns and solutions; drawing conclusions quickly and confidently, often to the detriment of the patient. Damage to the left PFC will allow the right PFC system – which supports the encoding and processing of ill-structured representations that facilitate lateral transformations – to have more impact. If the right PFC system remains totally unchecked, one would expect these patients (with left PFC lesions) to have enormous difficulty in articulating details and arriving at decisions.

The FLLH is not unrelated to ideas advanced by Beeman (1993) and Goldberg (2001). Beeman (1993) has developed a notion of coarse coding and fine coding of linguistic representations in the right and left hemispheres, respectively. Goldberg (2001) differentiates between left and right prefrontal cortex in terms of processing routine information (left PFC) and novel information (right PFC).

### EVIDENCE FOR LEFT PFC SYSTEM

Research with split-brain patients provides considerable evidence for the left hemisphere component of this hypothesis (Gazzaniga and Smylie, 1984; Gazzaniga, 1998, 2000). In one classic experiment (Gazzaniga, 1998), a split brain patient was presented with a picture of a winter scene projected to the right hemisphere (i.e., left visual field) and a picture of a chicken claw projected to the left hemisphere (i.e., right visual field). The patient must then select two related pictures, one picture with each hand, from an array of other pictures. The patient’s left hand points to a shovel (because the right-hemisphere, controlling that hand has seen a snow-covered winter scene) and the right-hand points to a chicken (because the left hemisphere, controlling that hand, has seen the chicken claw). When the patient is asked to explain why his left hand (guided by the RH) is pointing to the shovel, the left hemisphere (dominant for language) has no access to the information about the winter scene seen by the RH. But instead of responding “I don’t know” he responds by noting that the shovel is required to clean the chicken coop. These and similar findings have led Gazzaniga (1998, 2000) to postulate the existence of a “left hemisphere interpreter,” a pattern completion system that is compelled to connect bits of incomplete information and make sense of the world. It cannot tolerate indeterminacy or uncertainty. It must make connections, even though premature and flawed. This split-brain patient literature, however, limits the role of the RH to little more than visual organization (Corballis, 2003).

Gazzaniga’s (1998, 2000) conclusion about the dominance and role of the left hemisphere interpreter has been reinforced by recent neuroimaging studies showing strong left PFC involvement in a range of cognitive processes including hypothesis generation (Wolford et al., 2000), deductive reasoning (Goel, 2007; Prado et al., 2011), inductive reasoning (Goel and Dolan, 2004; Reverberi et al., 2005), and decision-making (DeNeys and Goel, 2011). In some of these tasks, such as deductive reasoning, the argument are determinant (though see below) and the conclusion are entailed by the premises. In the inductive reasoning tasks, while the argument itself is indeterminate, participants automatically make assumptions to eliminate the indeterminacy (as in the chicken claw and shovel example above).

### EVIDENCE FOR RIGHT PFC SYSTEM

Data supporting an extended role of the right PFC are harder to come by, largely because most neuropsychological tasks are well-structured and thus minimize the engagement of this system. But based on recent studies, I want to suggest that right PFC plays a critical but selective role in situations where the problem space (a) is very broad and poorly constrained, (b) contains misleading/conflicting information, and (c) contains insufficient information to determine the conclusion. These are all hallmarks of real-world problems.

One can get a glimpse of this role of right PFC even in well-structured tasks. For example, broadening the search space on scrambled word tasks by widening semantic categories words can belong to (e.g., “make the word ‘knife’ with IKFEN”; to “make a word for a kitchen utensil with IKFEN”; to “make a word with IKFEN”) reduces task constraints, broadens the problem space, and selectively engages right PFC (Vartanian and Goel, 2005).

Even in a classic “left hemisphere” task like logical reasoning, where participants are presented with transitive arguments (e.g., Mary is taller than John; John is taller than Angie; Mary is taller than Angie) and must determine if the conclusion follows from the premises, a recent study shows that patients with left PFC lesions are selectively impaired in trials with complete information (i.e., determinate trials; e.g., A > B, B > C, A > C and A > B, B > C, C > A), while patients with right PFC lesions were selectively impaired in trials with incomplete information (i.e., indeterminate trials; A > B, A > C, B > C; Goel et al., 2007). This patient study demonstrates a double dissociation across the two hemispheres along the dimension of determinacy, and provides strong evidence for the hypothesis. Neuroimaging studies reveal similar results (Goel et al., 2009; Brzezicka et al., 2011).

There are also data on “defocused attention” tasks from the cognitive creativity literature. A number of brain imaging studies do report RH involvement (either prefrontal cortex or superior temporal gyrus; Jung-Beeman et al., 2004; Kounios et al., 2008; Kounios and Beeman, 2010). However, the results across numerous studies do vary. A number of studies show bilateral frontal lobe involvement (Kounios et al., 2006; Aziz-Zadeh et al., 2009), while at least one highlights left PFC involvement (Luo et al., 2004). I suspect the variable results are a function of the varying task requirements across studies. The bilateral PFC results are not inconsistent with the frontal lobe lateralization hypothesis. The claim is not that one or the other hemisphere is required for a particular task, but that participation of the right PFC is necessary to successfully deal with ill-structured situations. This does not preclude the participation of the left PFC.

It is challenging to carry out experimental studies with ill-structured problems, and even more so within the constraints of brain imaging paradigms. Nonetheless, there are several attempts within the literature. Kowatari et al. (2009) carried out an fMRI study in which novice and experienced designers were asked to “think about new designs” for pens. The outputs were evaluated on a “good design award criteria” scale. Their main finding was that the design task involved activation in right inferior frontal gyrus, right inferior parietal cortices, bilateral inferior temporal cortices, and bilateral hippocampus. A comparison of left and right hemisphere activation revealed that the right PFC and parietal cortex showed significantly greater activation then left PFC and parietal cortex, particularly in the experts. Furthermore, they carried out a correlational analysis between individual originality scores (based on a “good design award criteria”) and the BOLD signal changes and found a significant correlation between the originality scores and the left minus right PFC BOLD signal, but not with right PFC or left PFC BOLD signals *per se*. This intriguing finding supports our contention that interaction between left and right PFC may be critical for good design solutions.

Ellamil et al. (2011) had university graphic design students engage in generate and evaluate cycles of book cover illustration design. These participants would have had considerable domain specific knowledge about graphic design. They were given verbal descriptions of books and allowed 30 s to generate an idea on a graphic tablet and 20 s to evaluate it, while undergoing fMRI scanning. They report that creative generation was associated with recruitment of medial temporal lobe regions while creative evaluation was associated with bilateral prefrontal and posterior regions. They suggest the following explanation as to why their task did not activate right prefrontal cortex in the generation condition: “the preferential recruitment of the medial temporal lobe during generation in the present study suggests that the participants, who were highly skilled art students exercising their abilities in a familiar task, may have generated ideas without the need for set shifting or dramatic conceptual reorganization, thus not requiring right PFC involvement.” Thus, while not supportive of the FLLH, these results are not necessarily inconsistent.

Perhaps the most directly relevant neuroimaging study to our topic is the architectural design task by Gilbert et al. (2009). This study used a clever task involving a plan drawing of a conference room (with a door, two windows, and a screen on one wall) and plan drawings of tables and chairs placed outside the room. There were two tasks. In the first, non-design problem-solving task, normal healthy individuals were asked to arrange the furniture in the room such that the following five constraints were satisfied: “(i) the two tables face each other; (ii) the long table is parallel to the screen; (iii) the participants can see each other; (iv) one participant cannot see the screen; (v) all the furniture is used.” In the second version of the task (the design task) the drawing stimuli were the same but the participants were asked to organize the tables and chairs in the conference room so that: “(i) the room is spacious; (ii) the room enables collaboration; (iii) the participants can see each other; (iv) all participants can see the screen; (v) you may use any of the furniture you like”. The second task involves greater degrees of freedom and interpretation than the first task and captures some of the interesting differences between well-structured and ill-structured problems discussed above. Participants indicated their solutions by moving and positioning the furniture pieces using a trackball system. They took on average longer to complete the design task (36 s) than the non-design task (31.8 s). The main result of the study was that the design condition was associated with greater activity in right dorsolateral prefrontal cortex compared to the non-design condition. A similar conclusion was reached in the two patient studies discussed below.

About 15 years ago I had the rare opportunity to test an accomplished, Yale educated, architect (Patient PF) who had the misfortune of suffering a right frontal parasagittal meningioma (Goel and Grafman, 2000). When I met PF he was bright, intelligent, and extremely articulate. Despite the pathology, his IQ and memory scores remained in the excellent range. But despite this, his personal and professional life had fallen apart and he found himself back living at home with his mother in his mid-50s. Since he was an architect we set him the task of redesigning our lab space. We asked another age and education matched professional architect to act as the control. A verbal protocol analysis methodology was employed (Ericsson and Simon, 1993).

**FIGURE 6:**
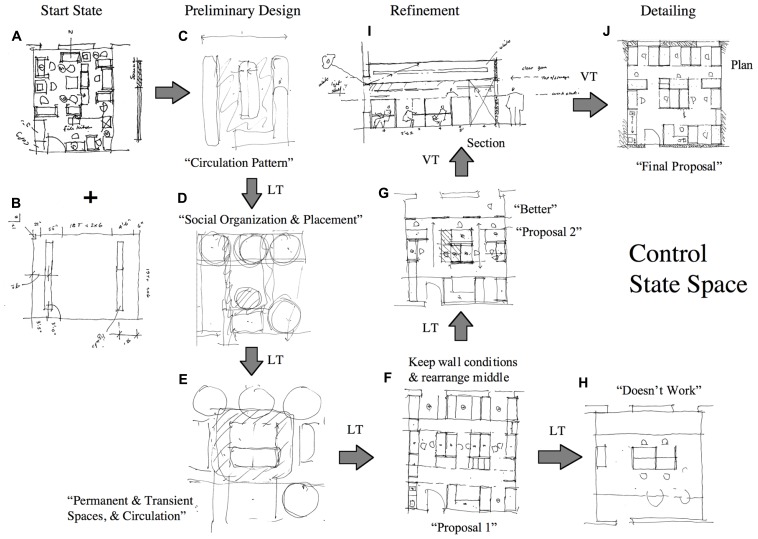
**Control subject’s state space and transformation functions.** Drawings **A** and **B** constitute the problem start state. The drawings **I** and **J** constitute the final (goal) state. Drawings **C** through **G** constitute the intermediate states. LT indicates a lateral transformation. VT indicates a vertical transformation. See text.

**Figure 6** depicts the solution space of the normal control subject. There are nine states including, a start state (A,B), a goal state (J) and seven intermediate states (C–I). The starting state is drawing (A) of the existing lab space and the accompanying measurement drawing (B). Three of the intermediate states belong to the preliminary design phase (C–E); four belong to the refinement phase (F–I); and the final state belongs to detailing (J). The preliminary design states are all quite abstract. He is not transforming walls and furniture. He begins by considering “circulation patterns” in drawing (C). This pattern constitutes his kernel idea. It is developed and transformed to deal with the issues of “social organization” (D) and “permanent and transient spaces” (E). The refinement drawings are structural. They depict concrete objects such as workstations, tables, doors, windows, and corridors. He transforms state (E) into a proposal (F; half way during the session) that he considers “reasonable.” However, he thinks the center condition can be improved. He therefore holds the perimeter conditions constant and transforms the center in drawing (H). He rejects drawing (H), returns to drawing (F) and transforms it into drawing (G). He is happy with the idea depicted by drawing (G). He now shifts gear and begins to detail and fine tune the proposal, first in section (I) and then in plan (J).

The movement from states (C) to (G) is underwritten by lateral transformations. The movement of states (G) to (I) and from (I) to (J) is underwritten by vertical transformation.

**FIGURE 7:**
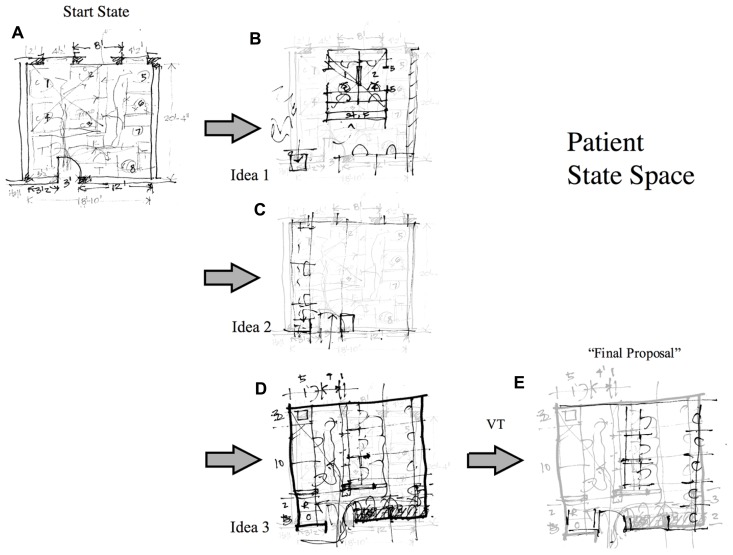
**Patient’s state space and transformation functions.** LT = lateral transformation; VT = vertical transformation. Drawings **B–E** were executed on transparent tracing paper on top of drawing **A**. Reproduced from Goel and Grafman (2000).

An analysis of the patient’s state space tells a very different story. There are five states in the patient’s solution space (**Figure 7**), a start state (A), a goal state (E) and three intermediate states (B–D). The start state drawing of the existing lab space (A) was completed by the patient from memory in the testing room. It is as detailed and accurate as the control’s drawing. The patient’s final state drawing (E) was completed during the refinement phase. The three intermediate drawings (B–D) were completed during the preliminary design phase. The first of these drawings (C) – the kernel idea – occurs two-thirds of the way into the session. Unlike the control subject, the patient is concerned with arranging furniture right from the start. But perhaps the most dramatic difference between the patient and control is that the patient’s three preliminary design drawings are fragmentary and unrelated.

Preliminary design sketches are, almost by definition, fragments of ideas. Designers do not typically generate several independent fragments and choose between them. They generate a single idea/fragment and develop it through transformations (lateral or vertical) to a point where it is complete and can be evaluated (Goel, 1995). The patient has made several (successful) attempts to generate idea fragments. But he is unable to develop and explore these ideas through the application of lateral transformations. Each of his preliminary drawings must be treated as independent idea/fragments. Indeed, he tries to articulate the difficulty he is experiencing as follows:

“You see, normally, what I would have, even as a student, I’d be – there would be sketches on top of sketches. And I could – it would be progressive. Here I seem to be doing several different thoughts on the same piece of paper in the same place, and it’s confusing me. So, instead of the one direction that I had at the beginning, I have three or four contradictory directions with not a kind of anchor to work from....”

The examination and testing of this patient suggested that he was unable to engage in lateral transformations because the representations utilized were too concrete and precise too early in the process. He could not abstract away from the particulars to consider the problem at a more abstract level. This is consistent with the claim that lesions to right prefrontal cortex may selectively impair patients’ abilities to manipulate abstract, indeterminate representations.

Design typically involves the specification of an artifact. Planning typically involves the specification of a sequence of actions. While planning and design are not identical they do overlap significantly at the cognitive level (Goel and Pirolli, 1989, 1992). Thus in another study we have used a Travel Planning Task to test the performance of patients with focal lesions to either left or right prefrontal cortex and posterior cortex, with that of normal controls (Goel et al., 2013). The advantage of such a task is that it does not require specialized knowledge. We all have some experience traveling and planning trips.

The task involved generating a plan for an American couple to spend a 1 week vacation in Italy. (The participants were Italian.) The task was constrained by time (1 week vacation), a budget ($3500), and differing interests of the couple. The participants were neurological patients with focal lesions to right prefrontal cortex, left prefrontal cortex, and posterior cortex. They were given a written problem statement followed by a pamphlet containing all the relevant information necessary for planning a trip, including a list of interests, flights, trains, car rental, accommodations, restaurants, attractions, maps, and distances between cities. They were encouraged to ask questions if any aspect of the task was unclear. The experimenter responded on behalf of the couple.

The task was administered in two parts, with no time restrictions. The first part required participants to read through the problem statement and the pamphlet and to complete a questionnaire designed to determine if they had understood the problem/task and were able to navigate through the information. If they were not able to answer the questions correctly, the experimenter helped them find the answers. This ensured that all participants had a similar level of understanding of the task before starting the actual planning phase. During the planning phase the participants were provided with blank paper and a diary divided into 7 days. They were requested to develop a daily schedule of activities, taking to consideration the constraints of time, interests, and money. The task was videotaped and participants were asked to “talk out loud” as they did the task (Ericsson and Simon, 1993).

It was found that patients with lesions to right prefrontal cortex generated substandard solutions compared to both normal controls and patients with lesions to left prefrontal cortex and posterior cortex. Examination of the underlying cognitive processes and strategies revealed that patients with lesions to right prefrontal cortex (driven by the intact left PFC) approached the task at an excessively precise, concrete level compared to normal controls, and very early locked themselves into substandard solutions relative to the comparison group. In contrast, the behavior of normal controls was characterized by a judicious interplay of concrete and abstract levels/modes of representations. Damage to the right prefrontal system impaired the encoding and processing of more abstract and vague representations that facilitate lateral transformations, resulting in premature commitment to precise concrete patterns, and hasty albeit substandard conclusions (because the space of possibilities had not been properly explored).

### SUMMARIZING AND CONTEXTUALIZING THE CASE FOR FLLH

The overall pattern of these data is consistent with the frontal lobe lateralization hypothesis. The hypothesis claims that left and right prefrontal cortex support different types of representations and cognitive processes. I agree with Gazzaniga (1998, 2000) that the left PFC abhors indeterminacy and is quick to impose some interpretation onto a situation. It is often dominant, preventing an adequate exploration of the problem space. Such a system is well-suited to support the precise, unambiguous, and determinate symbol systems required by inference mechanisms facilitating vertical transformations.

I contend that the critical feature of the right PFC’s role in the above studies is the inhibition of the left PFC and the accommodation of abstract, indeterminate representations of the task/situation. The former prevents the left PFC from forcing premature (often erroneous) interpretations of the task/situation while the latter supports associative mechanisms necessary to facilitate lateral transformations. Successful real-world problem-solving is a judicious balancing act between these two systems. When one system is damaged, the other will have excessive influence over behavior.

If this hypothesis is correct, it deepens and strengthens the case for the two mechanism model of design problem-solving offered above. The evidence for the nature of the left hemisphere systems ranges from the classical split brain patient studies of Gazzaniga (1998, 2000) and Gazzaniga and Smylie (1984) to more recent neuroimaging studies of well structured tasks, such as logical reasoning (Reverberi et al., 2005; Goel, 2007; Prado et al., 2011). The evidence for the role of the right PFC is a little bit more elusive, but largely because neuropsychologists have traditionally focused on well structured tasks. Nonetheless, a number of recent neuroimaging studies are highlighting the importance of right PFC in tasks where constraints are relaxed and the problem space is broadened (Vartanian and Goel, 2005). Other studies point to the importance of RH systems in classical creativity tasks involving “defocused attention” (Jung-Beeman et al., 2004; Kounios et al., 2008; Kounios and Beeman, 2010). Even indeterminate trials of deductive reasoning tasks result in robust involvement of right PFC ( Goel et al., 2007, 2009; Brzezicka et al., 2011). Moreover, several imaging studies specifically targeting graphic and architectural design tasks activate right PFC systems (Gilbert et al., 2009; Ellamil et al., 2011). Similarly, the two patient studies of architectural design and a travel planning task also highlight the necessity of right prefrontal cortex in successfully navigating ill-structured components of these tasks (Goel and Grafman, 2000; Goel et al., 2013).

The frontal lobe lateralization hypothesis give us a hemispheric asymmetry story superficially reminiscent of the left brain/right brain accounts that generated considerable academic interest in the 1960s and 1970s (Springer and Deutsch, 1998). These accounts were based on various divisions such as linguistic processes versus spatial processes, analytic versus synthetic processes, etc.. However, they have failed to survive in the academic literature because the data simply do not support these distinctions (though these accounts remain live and well in the popular psychology literature). The current proposal differs from the left brain/right brain account in three very important respects: (1) the current claim is about the differential roles of right and left prefrontal cortex (not each hemisphere). (2) It is based on a very different distinction having to do with certainty or determinacy of information being processed. This is independent of the linguistic/pictorial or analytic/synthetic accounts. (3) It is an interaction account whereby each hemisphere inhibits the other and biases certain types of processing. The default set up is probably a left hemisphere (PFC) bias or dominance. Individual differences in the inhibition of the left PFC may account for the relative ease or difficulty of engaging right PFC systems. In situations where the left PFC is lesioned, one would expect easier inhibition of the left PFC and more of the processing to be dominated by the right PFC. Where the right PFC is lesioned, one would expect the left PFC to be less inhibited and dominate the processing.

## CONCLUSION

I have sketched a preliminary framework of some cognitive and neuropsychological systems necessary to account for creativity in artifact design. The underlying theme is that an account of creativity should emerge from an account of real-world problem-solving. Creativity is unlikely to be a shining beacon to be discovered in the brain. It is more likely an outcome of the cognitive processes that allow us to engage in real-world problem solving. In terms of advancing our understanding of these systems, the basic claim is that a dual (or more likely, multiple) process model must be postulated. The two processes discussed here are associative mechanisms (facilitating lateral transformations) and inference mechanisms (facilitating vertical transformations), supported by indeterminate and determinate representations, respectively.

This account places a great deal of emphasis on the nature of the symbol systems that designers (in particular, architects) utilize, and the sequence in which they utilize them. An analysis of the symbol systems identifies determinacy as one critical dimension of variability and allows us to draw inferences about the nature of the cognitive machinery necessary to process them. We then appeal to the neuropsychological literature and argue that there are neuropsychological dissociations corresponding to the cognitive dissociations. This gives us a story connecting pre-theoretical intuitions about design activity, with cognitive theory and neuropsychological findings. The story is tentative, supported by some data, but subject to change and falsification as additional data are collected.

In terms of the question posed by the editor of this volume, “what if anything can neuroscience contribute to our understanding of creativity,” I think the answer is that, on its own, neuroscience’s current contribution must be modest and limited. However, in conjunction with cognitive theory, it has greater scope (Shallice and Copper, 2011). Most cognitive psychologists are happy to have cognitive theory inform neuropsychological research. So cognitive theory and data on real-world problem solving have taken neuropsychology beyond the left hemisphere interpreter and provided more realistic accounts of right PFC processes. However, the reverse interaction is less frequently appreciated. We must be equally ready to modify cognitive theory in light of neuropsychological data, in particular, data regarding double dissociations^6^. Recurrent patterns of double dissociation are indicative of causal joints in the cognitive system invisible in uninterrupted normal behavioral measures (Shallice, 1988). For example, the double dissociations along the lines of determinate (left PFC) and indeterminate (right PFC) representations (Goel et al., 2007), suggests that cognitive theory needs to be revised. The single process unitary models of human problem solving, whether they be the traditional information processing theory models (Newell, 1990) or the associative/subsymbolic models (Smolensky, 1988) must be reconsidered and replaced by dual or multiple mechanism models in light of the dissociations results. Such genuine, two-way interaction between cognitive psychology and neuropsychology will take us some way toward understanding human creativity and design problem solving.

## Conflict of Interest Statement

The author declares that the research was conducted in the absence of any commercial or financial relationships that could be construed as a potential conflict of interest.

## Footnotes

1 http://www.utzonoperahouse.com/memories.php

2 http://www.informationweek.com/it-leadership/steve-jobs-11-acts-of-vision/d/d-id/1100596

3 The term “contract documents” refers to the final technical drawings (i.e., blueprints) and accompanying linguistic specifications that specify the artifact to be constructed.

4 This is not to suggest that insight problems and divergent thinking problems are necessarily mutually exclusive. For example, the Remote Associates Task was developed by Mednick (1962) as a divergent thinking task. A number of researchers believe that it also has an insight component and use it as a insight task (Jung-Beeman et al., 2004).

5 There are also, of course, assumptions (explicit or implicit) about the cognitive/computational architecture.

6 Dissociation of brain functions: Brain lesions result in selective impairment of behavior. Such selective impairments are called dissociations. A *single* dissociation occurs when we find a case of a lesion in region *x* resulting in a deficit of function *a* but not function *b*. If we find another case, in which a lesion in region *y* results in a deficit in function *b* but not in function *a*, then we have a *double* dissociation.
